# Modernising grip dynamometry: Inter-instrument reliability between GripAble and Jamar

**DOI:** 10.1186/s12891-022-05026-0

**Published:** 2022-01-24

**Authors:** Sharah Abdul Mutalib, Michael Mace, Chloe Seager, Etienne Burdet, Virgil Mathiowetz, Nicola Goldsmith

**Affiliations:** 1 GripAble Limited, London, UK; 2 Bioengineering Department Imperial College of Science, Technology and Medicine, London, UK; 3 Occupational Therapy Program, University of Minnesota, Minneapolis, MN USA; 4 NES Hand Therapy Training, London, UK

**Keywords:** Hand strength, Grip strength, Dynamometry, Jamar, GripAble, Reliability, Occupational therapy, Physiotherapy, Hand rehabilitation, Outcome measure

## Abstract

**Introduction:**

Maximum grip strength (MGS) is a reliable biomarker of overall health and physiological well-being. Therefore, an accurate and reliable measurement device is vital for ensuring the validity of the MGS assessment. This paper presents GripAble, a mobile hand grip device for the assessment of MGS. GripAble’s performance was evaluated using an inter-instrument reliability test against the widely used Jamar PLUS+ dynamometer.

**Methods:**

MGS data from sixty-three participants (*N* = 63, median (IQR) age = 29.0 (29.5) years, 33 M/30 F) from both hands using GripAble and Jamar PLUS+ were collected and compared. Intraclass correlation (ICC), regression, and Bland and Altman analysis were performed to evaluate the inter-instrument reliability and relationship in MGS measurements between GripAble and Jamar PLUS+ .

**Results:**

GripAble demonstrates good-to-excellent inter-instrument reliability to the Jamar PLUS+ with ICC_3,1_ = 0.906 (95% CI [0.87—0.94]). GripAble’s MGS measurement is equivalent to 69% (95% CI [0.67—0.71]%) of Jamar PLUS+’s measurement. There is a proportional difference in mean MGS between the two devices, with the difference in MGS between GripAble and Jamar PLUS+ increasing with MGS.

**Conclusion:**

The GripAble is a reliable tool for measuring grip strength. However, the MGS readings from GripAble and Jamar PLUS+ should not be interchanged for serial measurements of the same patient, nor be translated directly from one device to the other. A new normative MGS data using GripAble will be collected and accessed through the software for immediate comparison to age and gender-matched subpopulations.

## Introduction

*Maximum grip strength* (MGS) is a ubiquitous objective outcome measure for delineating hand functions, including the severity of upper limb impairment, improvement after hand surgery and functional progress after rehabilitation (or lack thereof). A weak grip is associated with poor health-related quality of life (HRQoL) [1], and individuals with impaired grip strength pose an increased risk of having a heart attack, stroke, and cognitive loss [2, 3].

An accurate and reliable instrument for measuring MGS is crucial for ensuring the accuracy of grip assessment and the validity of the resulting clinical interpretation. MGS is typically measured using a handgrip device equipped with a force or pressure sensor – a *dynamometer*. The *Jamar* hydraulic hand dynamometer [4] is the gold standard device recommended by the American Society of Hand Therapists (ASHT) for measuring MGS [5]. Jamar is used in many studies for quantifying MGS, most notably in the well-known normative grip strength studies on adults and children by Mathiowetz et. al. [6, 7], which remain widely used by clinicians worldwide to compare patients’ grip strength.

Although Jamar hydraulic is accurate, reliable and has good-to-excellent test–retest reliability and excellent interrater reliability, it is also deemed outdated by many clinicians, as it is heavy at 680 g, has questionable robustness, insensitive for measuring low forces and requires regular recalibration. Therefore, many updated devices emerged that demonstrate good-to-excellent inter-instrument reliability with Jamar. These devices include Baseline [8], Rolyan [9], Grippit [10], MyoGrip [11] and Bodygrip [12]. Nonetheless, Jamar remains the gold standard, mainly due to heavy reliance and continued use of the aforementioned 1980s normative grip strength datasets. However, a recent study found that this normative data may no longer be valid, as grip strength has declined in recent decades. For example, the grip strength of Americans ages 20—34 has weakened dramatically relative to their 1980s counterparts [13]. This finding suggests that a new normative dataset is needed to consider the anthropometric and lifestyle changes in the past 40 years that may have altered the general population’s grip strength.

Whilst many previous studies used Jamar hydraulic to benchmark against new grip strength assessment devices, there is potential for significant reader error when using the Jamar hydraulic due to the dial only showing 2 kg increments [14]. Hogrel [11] also observed that Jamar hydraulic tends to overestimate grip force due to the inertial movement of the needle, which jumps slightly higher than the actual reading. The Jamar PLUS+ Digital is a digital analogue of the Jamar hydraulic which uses an electronic measurement principle based on a load cell force sensor instead of a hydraulic sensor [15]. The only other difference between the two Jamar devices is the weight (490 g vs 680 g) and small differences in external materials and finish. Despite being analogous, the inter-instrument reliability between the two Jamar models has been shown to be poor-to-moderate, with Jamar hydraulic measuring approximately 10% higher than Jamar PLUS+ [16]. This suggests that many device-specific factors contribute to differences in grip strength measurement including technological, as well as, size, weight, and material characteristics.

With an aim to modernise the current dynamometer, this paper presents the *GripAble hand grip device* – a new digital, accurate, sensitive and robust device for measuring grip strength. GripAble incorporates an electronic force measurement system with dual load cell sensors and connects wirelessly to a mobile device e.g. tablet, enabling users to perform objective MGS assessments. The addition of motion sensors enables the measurement of range-of-motion and device orientation allowing the user’s compliance to a standardised protocol, such as hand posture during MGS to be monitored.

According to Fess, the two most crucial criteria of any assessment device are (1) excellent measurement reliability across multiple sessions, examiners and devices and (2) high validity when compared against existing validated instruments. Fess further recommends (3) administrative instructions, (4) equipment criteria, (5) normative data, (6) instruction for interpretation and (7) a bibliography [17]. This paper aims to address the validity of GripAble by establishing the inter-instrument reliability compared to the Jamar PLUS+ . The Jamar PLUS+ was chosen due to the similarities in the underlying technology (i.e. digital force sensing) and the aforementioned limitations associated with the Jamar hydraulic device. We hypothesised that there would be an excellent inter-device reliability between Jamar PLUS+ and GripAble. However, larger diameter devices have been shown to yield lower grip force, as smaller intrinsic muscle groups will be recruited [18]. Therefore, considering the size difference between GripAble and Jamar PLUS+, we also hypothesised that there would be proportional differences in measurement output between the two devices, where the difference will be higher for individuals with higher MGS, with GripAble producing a small force output due to a relative increase in it's circumference.

## Methods

### GripAble hand grip device

GripAble is a device that comprises a dual load cell force-sensing mechanism that enables the handgrip to deform elastically when squeezed. GripAble has a sensitivity of up to 60 g and is accurate up to 2 kg of force [19]. It is also equipped with an IMU (accelerometer, gyroscope and magnetometer) for measuring wrist movement and device orientation [20]. The grip plate of GripAble can be set to either elastic or isometric modes [21] using a simple ‘locking’ button. It connects wirelessly via Bluetooth to an Android device with a custom app, which will read and record data associated with the user's hand grip and movement whilst using GripAble.

### Instruments

Two GripAble hand grip devices and two Jamar PLUS+ Digital Hand Dynamometers (referred to as Jamar+ hereinafter) were used for measuring MGS. Table 1 presents the feature comparison between GripAble and Jamar+ . All GripAbles and Jamar+ s were calibrated a maximum of two months prior to the start date of the data collection process and were otherwise unused to ensure a level playing field. The Jamar+ s were set on position 2 (second smallest handle position) following ASHT standardised position for measuring grip strength [5]. The GripAbles were used in isometric mode to match Jamar+ .

**Table 1:** Properties of GripAble and Jamar+ . Figures reprinted from Performance Health Sammons Preston users’ guide

Properties	GripAble	Jamar PLUS+ Digital
		
Weight (g)	240	490
Front-to-back depth (mm)	48 (isometric mode)	49 (position 2)
Side-to-side width (mm)	40	25
Circumference (mm)	141 (isometric mode)	128 (position 2)
Measurement units	kg or lbs	kg or lbs
Measuring modes	Elastic and isometric	Isometric only
Increments of measurement unit (kg)	0.1 (0—90)	0.1 (0—90)
Readings (digital/non digital)	Digital with mobile app integration	Digital readout
Calculations	Maximum value, mean, standard deviation, left–right ratio	Mean, standard deviation, coefficient of variation
Data tracking/recording	Automatic through mobile app	Not available/manual

### Participants

Sixty-three healthy participants (*N* = 63) aged between 16 – 80 were recruited for the study. Each participant was assigned an alphanumeric ID and randomly split into four different experiment groups: GripAble right first (GR), GripAble left first (GL), Jamar+ right first (JR), and Jamar+ left first (JL).

Participants were included if they had no diagnosed upper limb pathology, pain in the hand, wrist or forearm, or history of neurological disorder affecting the upper quadrant. All participants had a good comprehension of English to understand verbal instructions and experimentation documentation. Participants outside these criteria were excluded.

All participants gave informed consent before the study. The experiment was performed following the ethical standards laid down in the 1964 Declaration of Helsinki and was approved by the Imperial College Research Ethics Committee (ICREC) and Science, Engineering & Technology Research Ethics Committee (SETREC).

### Procedure

The study took place during the COVID-19 pandemic, i.e. August—December 2020. Therefore, all experiments were conducted outdoors, with a 2-metre distance between the investigator and the participant strictly followed as per NHS England COVID-19 guidelines. At the start of each session, the participant and investigator washed their hands thoroughly, and both devices were sanitised with isopropyl alcohol (IPA) wipes between participants.

**Fig. 1:**
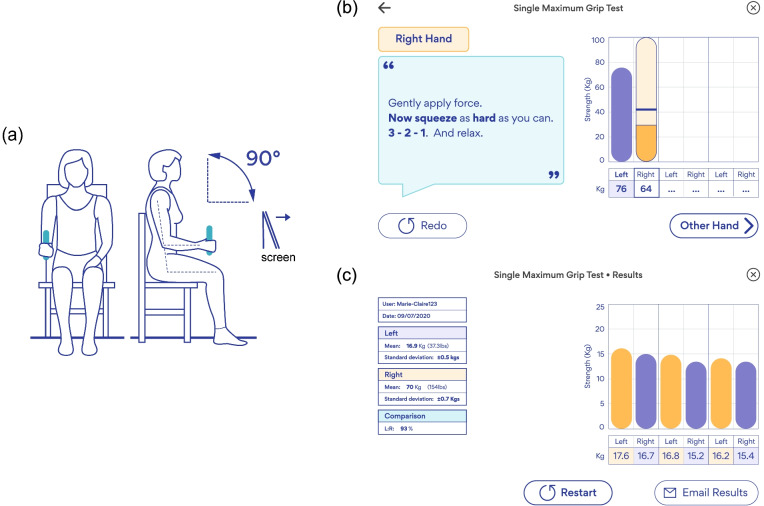
Single maximum grip strength test for measuring MGS: (**a**) The sitting posture of participants following ASHT guideline. The screenshots of GripAble’s app displaying (**b**) the single maximum grip strength test page and (**c**) the result page. These screens are investigator-only views

Each participant was positioned upright in a seated position on a chair with no armrests during the experiment. The investigator ensured that each participant maintained similar positioning at all times, which was standardised according to the ASHT guidelines [5], including legs uncrossed, the bottom of the spine positioned against the back of the chair with hips and knees positioned at approximately 90°, arm adducted, elbow flexed at 90°, forearm in neutral and wrist in comfortable 15° to 30° extension and 0° to 15° of ulnar deviation (Fig. 1a). Before starting, the investigator first demonstrated a single maximum grip test protocol. Verbal instruction was given, i.e. “Gradually put on the force. Now squeeze as hard as you can. 3, 2, 1, and relax” by the investigator during each measurement, which was also displayed on the GripAble app screen and only visible to the investigator.

Three MGS measurements were recorded from each hand, using each device (i.e. twelve measurements in total). The hand was alternated between trials (e.g. GripAble R-L-R-L-R-L, followed by Jamar+ R-L-R-L-R-L). A minimum 15-s rest was enforced between each measurement of the same hand and a minimum of three-minute rest between devices, as per ASHT guidelines. The measurement from the opposite hand was taken during the 15-s rest. Two investigators collected all measurements using the same sets of Jamar+ and GripAble devices throughout the study (i.e. one set for each investigator). All measurements taken using GripAble were calculated automatically by the GripAble app, and results were displayed to the investigator at the end of the test. The screen showing the GripAble app faced the investigators the entire time. Fig. 1b and 1c show screenshots of the GripAble app’s test pages displaying the test page and result page, respectively.

### Data analysis and statistics

The mean MGS of the three trials from each hand and device was used for data analysis, as per ASHT guidelines. The mean MGS of all participants from both hands (*N* = 126) were then tested for normality using a Shapiro–Wilk test. Statistical significance was calculated at a 95% confidence level (*p* < 0.05).

A zero-intercept linear fit using total least squares (TLS) was used to analyse the relationship between the measurements from GripAble and Jamar+ . Meanwhile, the inter-instrument reliability between Jamar+ and GripAble was tested using intraclass correlation coefficient (ICC) assuming average fixed raters, i.e. ICC_3,1_, which measures the inter-instrument reliability in terms of *‘consistency’* [22]. ICC values of < 0.5, 0.5 - 0.75, 0.75 - 0.9, and > 0.90 indicate poor, moderate, good and excellent reliability, respectively. The ICC was calculated across all participants, as well as separately for each gender, starting device order and starting hand order, for each device and hand.

A Bland and Altman plot was used to evaluate the MGS values measured using Jamar+ and GripAble. The plot visualises the differences in MGS (${D}_{GJ}$) between the two devices against their means (${\mu }_{GJ}$). An ordinary least square (OLS) regression analysis was performed on this relationship to the proportionality of relationship between these two terms. The regression of ${D}_{GJ}$ on ${\mu }_{GJ}$ can be described in the form of a *y*-intercept and a slope of the regression. A slope that is significantly different from zero (i.e. *p* < 0.05) suggests a proportional difference in MGS between Jamar+ and GripAble, where the difference increases (or decreases) as the mean increases. In this case, the limits of agreement are calculated using a regression approach for nonuniform differences [23, 24].

Statistical differences of participants’ age between genders (male vs female), between starting device orders (GripAble first vs Jamar+ first) and between starting hand orders (right first vs left first) were calculated using a Mann–Whitney U test. Statistical differences in MGS between genders (male vs female) was calculated using a Mann–Whitney U test, whilst between hands (right vs left) and devices (GripAble vs Jamar+) were calculated using Wilcoxon signed-rank tests. Finally, the influence of starting device and starting hand orders on MGS was also analysed using a Mann–Whitney U test, i.e. GripAble first vs Jamar+ first, and right hand first vs left hand first.

## Results

A Shapiro–Wilk test revealed a non-normal distribution of MGS measured using both GripAble and Jamar+ (*p* < 0.001 for both). Therefore, results are presented as median (interquartile range; IQR) and non-parametric tests were used for statistical analysis.

### Participants

Table 2 describes the demographic information of the participants (*N* = 63). Participants were predominantly self-reported right-handed (*N*_R_ = 57; *N*_L_ = 6). Mann–Whitney U tests revealed no significant difference in participants’ age between genders (male vs female), between starting device orders (GripAble first vs Jamar+ first) and between starting hand orders (right first vs left first) ( *p* > 0.05 for all three comparisons performed).

**Table 2:** Demographic information of the participants. Shown are the median (IQR) ages of the participants. The last column shows the results from Mann–Whitney U tests to calculate statistical differences in participants’ age between genders, between starting devices and between starting hands

Category	N	Age (years)	Statistical difference (*p*-value)
**All**	63	29.0 (29.5)	-
**Gender**			
* Male*	33	29.0 (29.0)	Not significant (*p* = 0.78)
* Female*	30	30.0 (29.5)	
**Starting device**			
* GripAble first*	32	35.0 (30.5)	Not significant (*p* = 0.77)
* Jamar*+ *first*	31	29.0 (26.0)	
**Starting hand**			
* Right first*	31	29.0 (25.5)	Not significant (*p* = 0.26)
* Left first*	32	33.0 (31.25)	

### Inter-instrument reliability between GripAble and Jamar+ 

**Fig. 2:**
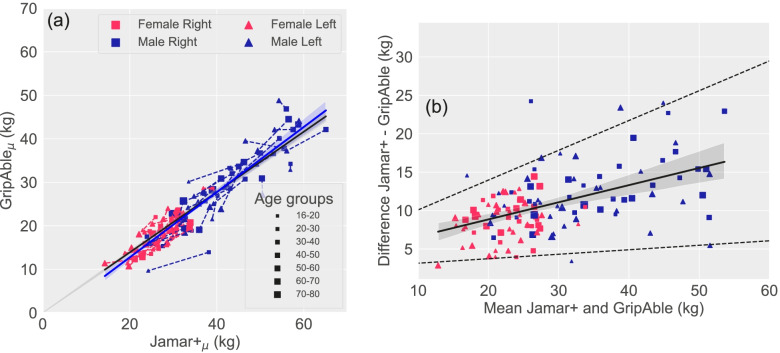
Inter-instrument reliability analysis between GripAble and Jamar+ dynamometers: (a) A regression line and scatterplot showing high linearity of the MGS measurements between GripAble and Jamar+ across participants. (b) Bland–Altman plot showing the differences in Jamar+ and GripAble measurements ${D}_{GJ}$ against their means ${\mu }_{GJ}$. The solid line is the statistically significant regression line between these two parameters, suggesting a proportional relationship where the difference increased as the mean increased. The upper and lower dashed lines are limits of agreement computed using the method outlined in Bland (1999) [23]

Figure 2a shows the mean MGS for each participant comparing Jamar+ (*x*-axis) to GripAble (*y*-axis). The right (square markers) and left (triangular markers) hands are shown as connected points. Age and gender are indicated by the marker size and colour, respectively. A zero-intercept linear fit using TLS revealed that GripAble’s measurement output is equivalent to 69% (95% CI [68—71]%) of Jamar+ ’s measurement output.

Figure 2b shows a Bland-Altman plot visualising the differences in MGS between GripAble and Jamar+ , ${D}_{GJ}$ as a function of their means, ${\mu }_{GJ}$. The limits of agreement are shown as two dotted lines. Jamar+ tended to read higher MGS than GripAble, and this difference was not fixed in absolute value but proportionally increased when MGS increased. This proportionality can be expressed with a *y*-intercept of 4.39 (95% CI [2.36—6.42]) and a slope of 0.22 (95% CI [0.16—0.29]) (*p* < 0.001 for both). The overall regression was statistically significant (*R*^*2*^ = 0.26, *F*(1, 126) = 43.98, *p* < 0.001).

Inter-instrument reliability was tested using intra-class correlation (ICC) assuming average fixed raters, i.e. ICC_3,1_ The overall ICC value was computed across all data collectively, and an overall ICC of 0.909 (95% CI [0.87—0.94]) indicates a good-to-excellent inter-instrument consistency between the two devices. The ICC was also calculated separately for each gender, starting hand order and starting device order. The full ICC results are shown in Table 3.

**Table 3:** Intraclass correlation coefficient (*ICC*) analysis between GripAble and Jamar+ . Overall, results show good-to-excellent consistency between GripAble and Jamar+

Category	Hand	# Datapoints	ICC	ICC Confidence interval 95%
**Overall**				
	Both	126	0.909	[0.87—0.94]
	Right	63	0.920	[0.87—0.95]
	Left	63	0.898	[0.84—0.94]
**Gender**				
* Male*	Right	33	0.899	[0.81—0.95]
	Left	33	0.887	[0.78—0.94]
* Female*	Right	30	0.859	[0.72—0.93]
	Left	30	0.811	[0.64—0.90]
**Starting device**				
* GripAble first*	Right	32	0.937	[0.87—0.97]
	Left	32	0.919	[0.84—0.96]
* Jamar*+ *first*	Right	31	0.903	[0.81—0.95]
	Left	31	0.878	[0.76—0.94]
**Starting hand**				
* Right first*	Right	31	0.894	[0.80—0.95]
	Left	31	0.899	[0.81—0.95]
* Left first*	Right	32	0.951	[0.90—0.98]
	Left	32	0.893	[0.79—0.95]

### Influence of hand, gender, starting device order and starting hand order on MGS

Table 4 summarises the descriptive data on the mean MGS out of three trials from all participants using both GripAble and Jamar+.

**Table 4:** The descriptive data on MGS measurements out of three trials from all participants (*N* = 63), presented as median (IQR). Shown are the MGS measurements by hand and gender, as well as by starting device/starting hand order, for both GripAble and Jamar+

Category	# Datapoints	GripAble mean MGS (kg)	Jamar+ mean MGS (kg)
**Overall**	126	21.48 (10.95)	31.35 (13.40)
**Hand**			
* Right hand only*	63	21.87 (12.30)	31.77 (15.43)
* Left hand only*	63	21.17 (10.03)	30.50 (12.18)
**Gender**			
* Male only*	66	30.73 (14.73)	42.83 (18.83)
* Female only*	60	18.85 (6.06)	28.72 (6.18)
**Starting device**			
* GripAble first*	64	21.92 (10.81)	31.30 (12.89)
* Jamar*+ *first*	62	20.07 (9.68)	31.28 (14.09)
**Starting hand**			
* Right first*	62	22.5 (10.83)	31.87 (12.36)
* Left first*	64	20.15 (11.04)	30.75 (13.33)

**Fig. 3:**
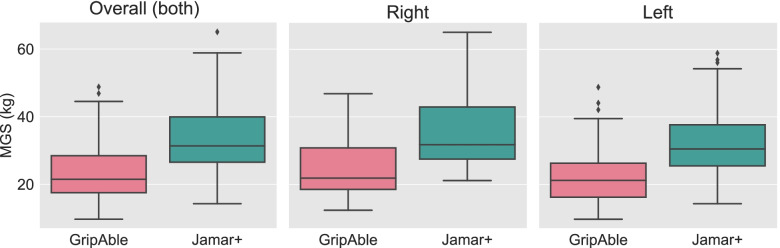
MGS for both hands, as well as for right and left hands measured using GripAble vs Jamar+ . There were significant differences between MGS measured using GripAble vs Jamar+ for all three comparisons performed (Wilcoxon signed-rank test; *p* < 0.001 for all)

Figure 3 visualises the mean MGS from each device and hand from the three trials. Wilcoxon signed-rank tests revealed significant differences between the overall MGS, as well as right hands’ and left hands’ MGS between GripAble and Jamar+ (*p* < 0.001 for all three comparisons).

**Fig. 4:**
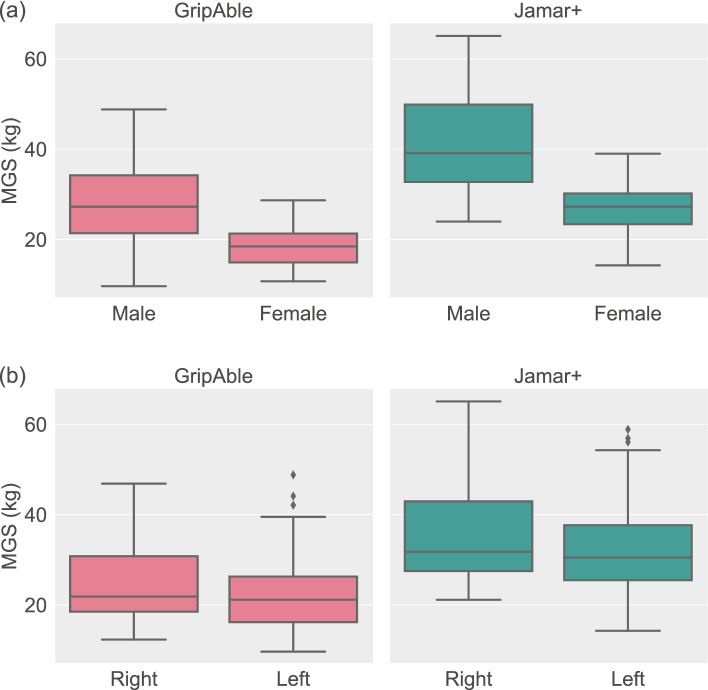
Influence of gender and hand on MGS. Boxplots showing the differences in grip strength between (**a**) male vs female and (**b**) right hand vs left hand, measured using GripAble (left column) and Jamar+ (right column). There were significant differences in MGS across all four comparisons performed (Mann–Whitney U test for gender comparison, Wilcoxon signed-rank test for hand comparison; *p* < 0.001 for all)

Figure 4 visualises the influence of gender and hand on MGS. A Mann–Whitney U test revealed significant differences between males and females (*p* < 0.001 for both GripAble and Jamar+), whilst a Wilcoxon signed-rank test revealed significant differences between right and left hands (*p* < 0.001 for both GripAble and Jamar+).

A Mann–Whitney U test revealed no significant differences in MGS between participants that started with GripAble first or Jamar+ first (*p* = 0.45 for GripAble measurements, *p* = 0.97 for Jamar+), or between participants that started with the right hand first or left hand first (*p* = 0.28 for GripAble measurements, *p* = 0.29 for Jamar+), suggesting that the starting device and starting hand orders did not influence the MGS measurements.

## Discussion

The MGS measurements from sixty-three participants using GripAble and Jamar+ show that GripAble has an overall good-to-excellent inter-instrument reliability with Jamar+ , with an overall ICC of 0.909 (95% CI 0.87—0.94). GripAble’s force output is equivalent to 69% (95% CI [0.67—0.71]%) of Jamar+ ’s force output. Jamar+ tended to read higher MGS than GripAble. The difference was not fixed in absolute value but proportionally increased with higher MGS.

The difference in readings between the two devices is not unique to this study. In a previous study by Hogrel, Jamar was 14% higher than MyoGrip, and the difference proportionally increased with higher MGS [11]. Although they attributed the difference to the inertial movement of Jamar’s needle, we hypothesised that *physical* differences between GripAble and Jamar+ , including circumference and weight, play a more significant role. Specifically, a larger diameter device such as GripAble encourages flexion at metacarpophalangeal (MCP) joints and therefore recruits smaller intrinsic muscles. In contrast, the smaller Jamar+ encourages intrinsic minus grip pattern with the proximal interphalangeal joints predominantly active, increasing the force output for the same individual. Moreover, GripAble is also nearly half the weight of Jamar+ , which may have further contributed to the difference.

*Psychological* factors could also play a part. Jamar+ is made of metal and plastic, whereas GripAble is made entirely of plastic. The material difference and the lower weight of GripAble may have given the impression of it being more fragile, causing the participants to be more reserved from exerting maximal grip force on the device. Therefore, verbal reinforcement may be needed for future grip assessment studies to encourage the user to squeeze maximally when using GripAble.

Although Jamar is considered the gold-standard device for measuring MGS, it is outdated and needs updating to reflect evolving digital mobile health (*mHealth*) technology trends. With the recent global COVID-19 pandemic, the rehabilitation paradigm has shifted from inpatient to outpatient venues, including remote management using virtual platforms. Presently, clinicians who are managing their patients remotely need to rely on self-reported grip strength capabilities. Patients may benchmark against a change in their ability to carry out ADLs, which is not sensitive or objective. GripAble’s custom mobile software platform provides an objective measurement of MGS completely remotely. Such a feature allows patients to regularly test their grip strength whilst allowing therapists to monitor and assess patients both accurately and reliably over time without their physical presence. Moreover, the integrated sensors within GripAble and software can provide researchers with additional information on the exact positioning of the device and hand postures during MGS assessment. For example, the ASHT guideline for measuring MGS recommended that the device be held vertically and stably. Using the data provided by the motion sensors within GripAble, it would be possible to explore user compliance to this guideline.

Hand grip is vital for activities of daily living, and an extensive body of research has shown that it is a reliable biomarker of overall physical health and wellbeing, including predicting mortality and morbidity risks [25]. Nevertheless, the true impact of impaired grip strength on one's functional performance of ADLs is poorly understood. Most commonly, grip strength is only assessed as a single maximum effort, which only measures capability during one short instance. In functional activities, grip needs to be employed in many different ways, such as sustained over a more extended period or repeatedly applied. GripAble provides a range of grip tests, including but not limited to grip endurance, sustained gripping, grip in various forearm rotational positions [26], rapid exchange [27] and sine wave grip accuracy tests [28], to allow for a more holistic view of hand function. Ultimately, this opens the opportunity for a deeper understanding of the association between grip and functional performance, where the multiple aspects of grip may uncover such associations.

## Conclusion

This study provides evidence of GripAble’s good-to-excellent inter-instrument reliability and consistency to the Jamar+ . There was a proportional bias between the two devices, where the differences in MGS readings between the two devices increased as their means increased. Therefore, whilst measurements from both devices can be considered valid, the MGS readings from these two devices should not be interchanged for serial measurements of the same patient, such as when comparing pre and post-intervention, nor be translated directly from one device to the other.

GripAble can be used clinically as a dynamometer whilst providing additional software benefits, including the display of postural and procedural instructions, standardised narrative and automated result computation and analysis. Furthermore, integrating a dynamometer into an accessible multifunctional training device gives the potential for objective assessment both in the clinic and remotely for home users. Further studies investigating the other facets of grips, as well as test–retest and inter-rater reliability are needed. Updated device-specific normative values for age and gender subsets are also needed and, once available, can be integrated into the software for immediate comparison.

## Data Availability

The data supporting the findings of this study are available within the article.

## Abbreviations

MGS: Maximum grip strength
ICC: Intraclass correlation coefficient
IQR: Inter-quartile range
ADL: Activities of daily living
ASHT: American Society of Hand Therapists
IMU: Inertial measurement units
